# “We need to talk to each other”: Crossing traditional boundaries between public health and occupational health to address COVID-19

**DOI:** 10.3389/fpubh.2022.1046628

**Published:** 2022-12-06

**Authors:** Pamela Hopwood, Ellen MacEachen, Shannon E. Majowicz, Samantha B. Meyer, Joyceline Amoako

**Affiliations:** School of Public Health Sciences, Faculty of Health, University of Waterloo, Waterloo, ON, Canada

**Keywords:** public health systems research, population surveillance, occupational exposure, occupational health, interdisciplinary, COVID-19

## Abstract

**Introduction:**

This study examined how public health (PH) and occupational health (OH) sectors worked together and separately, in four different Canadian provinces to address COVID-19 as it affected at-risk workers. In-depth interviews were conducted with 18 OH and PH experts between June to December 2021. Responses about how PH and OH worked across disciplines to protect workers were analyzed.

**Methods:**

We conducted a qualitative analysis to identify Strengths, Weakness, Opportunities and Threats (SWOT) in multisectoral collaboration, and implications for prevention approaches.

**Results:**

We found strengths in the new ways the PH and OH worked together in several instances; and identified weaknesses in the boundaries that constrain PH and OH sectors and relate to communication with the public. Threats to worker protections were revealed in policy gaps. Opportunities existed to enhance multisectoral PH and OH collaboration and the response to the risk of COVID-19 and potentially other infectious diseases to better protect the health of workers.

**Discussion:**

Multisectoral collaboration and mutual learning may offer ways to overcome challenges that threaten and constrain cooperation between PH and OH. A more synchronized approach to addressing workers' occupational determinants of health could better protect workers and the public from infectious diseases.

## Introduction

The SARS-CoV2 (hereafter, COVID-19) pandemic led to jurisdictions rapidly developing approaches to control disease spread. Despite advanced planning for the management of infectious disease and health emergencies stemming from previous public health (PH) risk events, such as SARS-CoV-1 (1), gaps appeared in areas such as data access, surveillance and consistent risk communication (2). The Public Health Agency of Canada stated that new connections across disciplines—which we refer to as multisectoral collaboration—had been made during the pandemic and recommended that these should be retained (2), though specific ways in which multisectoral collaboration might occur, and the challenges faced, remain undocumented.

Multisectoral collaboration has been considered in other PH-related studies (3) although, to our awareness, this has not been explored with consideration of occupational health (OH), PH and COVID-19. By PH, we refer to federal and provincial government agencies that focus, at the population level, on preventing disease and injuries, responding to public health threats, and promoting good physical and mental health. By OH, we refer to activities usually carried out by provincial Ministries of Labor that are focused on workplace safety and prevention of workplace accidents and illness. The involvement of various levels of government and a range of expertise that includes transdisciplinary knowledge is recognized as important for overcoming challenges in implementing and sustaining successful partnerships (4). Previous literature about multisectoral collaboration has noted complex PH issues have benefitted or been hindered by factors such as culture, structure, and leadership (5). Key strategies for success have included clear shared goals and roles, accountability measurements, and involvement of all sectors from the planning stage (5). Healthy school programs across Canada demonstrated the potential for longstanding and widescale impact through cross-sector engagement between PH and education systems (6). A 2022 systematic review examining governance during public health emergencies found it is important to document how policy actors communicate and coordinate to learn effective strategies for policy implementation (7).

Overlapping PH and OH issues related to COVID-19 were evident in workplaces, yet PH and OH responses were often siloed. The health risk from COVID-19 affected many industries, although low-wage workers, who were often public-facing, were disproportionately at risk (8). An Ontario study estimated that 12% of COVID-19 cases from April 1, 2020 to March 31, 2021 were linked to workplace outbreaks (9); however, these were only a portion of the cases that occurred in workplaces (others were attributed to community transmission and many were not reported at all). Embedded in occupation-based risk were class and racial disparities. For instance, in Toronto and Vancouver, the highest measure of inequality for COVID-19 positivity was among workers assessed as least able to work from home (often recent immigrants), while in Montreal the greatest inequality was among people who were visible minorities (10).

In this paper, we consider PH and OH COVID-19 measures reported by experts knowledgeable about their provincial settings. This study was important to explore how OH and PH experts perceived multisectoral work during the pandemic, and their suggestions about what worked well and what could be improved. Our study examined the following question: what can be learned from how these two sectors work together and separately to address occupational determinants of health affecting workers during the COVID-19 pandemic? Specifically, we investigated how PH and OH worked in relation to each other during the pandemic in Canada's four most populated jurisdictions: Alberta, British Columbia, Ontario and Quebec.

## Methods

### Study design, sample, and recruitment

This qualitative study is based in situational analysis and particularly, situational mapping whereby aspects of the situation and the relations between these aspects (e.g., policy or organizations) are analyzed (11). Our analysis drew on key informant (KI) expertise and utilized a policy-topics framework to identify Strengths, Weaknesses, Opportunities and Threats (SWOT) in multisectoral collaboration and prevention approaches. Strengths and weaknesses are conceived of as internal whereas opportunities and threats are considered largely external to the organization(s) at the center of analysis (12).

In-depth interviews were conducted with experts on how OH and PH sectors managed separately and collaboratively during the pandemic. We interviewed 18 key KIs, who were leaders in their areas of areas of labor, OH, employment law, and PH across Canada's four most populous provinces: British Columbia, Alberta, Ontario, and Quebec. These provinces were selected because they provided useful contrasts between OH and PH systems due to different structures and policies. Quebec and British Columbia have work-related injury and illness prevention and workers' compensation within the same organization: Commission des normes, de l'équité, de la santé et de la sécurité du travail in Quebec, and WorkSafe BC in British Columbia. Prevention and compensation are under separate organizations in Ontario where there is the Workers Safety Insurance Board, and The Ministry of Labor, Training and Skills Development, and in Alberta where there is the Workers Compensation Board of Alberta and the Occupational Health and Safety government branch.

Internet searches and government and industry organizational charts, publications and reports, news media and LinkedIn were used to identify key practitioners and policy experts with close knowledge of OH and PH policy, including relating to at risk workers. Using a purposive sampling strategy as we aimed for representation from PH, OH, and employment policy experts across the four jurisdictions. We recruited from the identified pool through cold calls, email, web forms and snowball/chain referral. We reached out to 117 contacts; some of these were professional and other organizations that, in turn, sent recruitment materials to an internal workforce or membership. Despite significant recruitment attempts, we did not secure PH expert interviews in Alberta and British Columbia, nor a worker advocate in Quebec. KIs had 1 to 25 years' experience (average 10 years) in PH and OH or policy.

Our final sample included government employees, lawyers from independent firms and legal aid organizations, union representatives, employees of non-profit organizations specializing in policy research and recommendations, and of non-profit social and worker advocacy organizations (Table 1).

**Table 1:** Sample areas of expertise per province.

**Sample: Area of expertise**	**Alberta (P16–18)**	**British Columbia (P11–15)**	**Ontario (P1–P6)**	**Quebec (P7–P10)**
Occupational health	1	2	2	1
Public health	-	-	2	2
Worker advocate	1	2	1	-
Employment standards	1	1	1	1
Total	3	5	6	4

Provides a count of the key informants in each province and their area of expertise.

### Data collection and analysis

Semi-structured, in-depth interviews, lasting 45 min to 1 h were conducted by the lead author between June and December 2021. Most were *via* Microsoft Teams; some were by phone when the participant preferred. Questions covered how/if PH and OH worked together during the pandemic, the scope of PH and OH operations, and protections and gaps for the health of workers.

Interviews were audio recorded and transcribed verbatim. Detailed notes capturing contextual issues and new analytic insights were written immediately following each interview, and then discussed by team members. Interview transcripts were reviewed for accuracy and uploaded to NVivo (13).

A framework with a-priori topic areas (Table 2) related to our research question was developed.

**Table 2:** Framework topics.

**Topic**
Public health programs/measures
Occupational health and safety
Workers' compensation
Employment insurance
Employment standards
CERB/CRB
Government structure/jurisdiction issues
How policy/practice affects employers
How policy/practice affects workers
Union role
Key policy weaknesses
OH-PH Collaboration

This table provides a list of topics determined a-priori that were included in the framework.

Interviews, field notes and emerging topics for the framework were discussed at weekly team meetings as data familiarization. As patterns began to appear (e.g., “Siloed legislation”) additional topics were added to the framework (14). The framework was used to develop a codebook.

The coded data were used for in-depth descriptions of each policy and thematic area and were used to compare the findings from the four provinces. With comparative data, we conducted a SWOT analysis as our final interpretation and mapping step (14). Documentary data were also gathered as topics arose, concurrent with interviews and analysis, to deepen and contextualize KI information. These materials, including employment standards/code, workers' compensation acts, and law journal legal interpretations, provided additional insight into work and health policy.

This research was approved by the Human Research Ethics Board at *[University blinded for review]*.

## Results

Our findings are distilled into themes under the headings of our SWOT analysis. In terms of *strengths*, we found novel collaboration occurred between PH and OH. However, *weaknesses* in organizational elements (e.g., siloes) impacted how PH and OH government bodies functioned in the context of the pandemic. This resulted in sectoral boundaries that sometimes impeded potential for PH and OH to improve health conditions. Third, we found there were several *opportunities* for further collaboration. Finally, our results identified *threats* which could be barriers to working together to address issues, and potential for ongoing gaps in worker protection.

### Strengths

OH and PH sectors were described by KIs as having shared interest in health and safety risks and in prevention activity, which intersected within workplaces with infectious disease, such as COVID-19. For example, an Ontario PH KI described how their PH inspectors included work organization issues when investigating how people might have been exposed to COVID-19 (see Table 3, quote iii). In Ontario, KIs and documents both explained that PH became involved in workplaces in response to disease outbreaks, with COVID-19 outbreaks considered as two or more laboratory-confirmed cases where workers on the same shift or work area plausibly contracted the disease at work (15).

**Table 3:** Strengths key points identified from quotes about PH-OH pandemic response and KI province.

**Quote no**.	**Prov**.	**Quote**	**Key points for collaboration**
**i**	British Columbia	“It's been a unique year and a half in terms of linking up with public health …. the state of emergency in COVID-19 has meant that we were very much engaged in doing a lot of heavy lifting for PH in terms of getting workplaces to have COVID-19 safety plans and put in controls and do all that, so …. We've definitely grown a lot into that area …. We've seen …. the benefit in the employers dealing with disease better; not just, you know, your typical occupational disease which …depends on your workplace, but this is … publicly contracted, communicable disease, so it's something we're going to continue to be involved in. So, it's a bit of a new area, a new way of looking at things” (P15, OH Expert, British Columbia).	New interaction between OH and PH OH inspectors assisted with implementation and education about COVID-19 safety plans OH saw improvement in how workplaces manage communicable disease
**ii**	Quebec	“…one of the early mandates during the pandemic, from the health ministry, was to put together … a work group on COVID and occupational health … to produce the recommendations for workers and for sectors.… It was just a need that was expressed by the people working on the COVID response to say, ‘We need to talk to each other', and one example …was regarding the recommendations for schools, the type of masks. So, the group working on the general population measures and the group working the recommendations for workers spoke to each other to … say … ‘Recommend the same type of mask for everyone in the schools, whether they're the students—or the teachers, the workers”' (P7, PH expert, Quebec)	Ministry of Health directive PH and OH identified need to work together Ensured consistency in recommendations for mask types
**iii**	Ontario	“…they'll have a huge checklist of things that they will go through and then they'll see, ‘Okay, we had our break together for 30 min and our chairs are two feet apart.' So, then they'll go through and make suggestions, ‘Okay, this is how you're going to decrease the risk of transmission amongst your staff …. Make sure that the staff is wearing mask when they're having the patrons come and pick up their takeout.' The source of transmission is … identified during the case investigation and then the PH inspector will actually go to that facility or that restaurant and go through their checklist and see: Where are the areas of risk and, what can be updated or corrected to decrease risk in the future?” (P3, Ontario, Public Health).	PH inspectors investigated the organization of work and workplace practices PH considered workplace areas of risk and provided risk mitigation strategies

This table provides KI quotes and describes key strengths identified from quotes about PH-OH pandemic response and KI province.

During the pandemic, KIs described how OH and PH collaborated to develop COVID-19 safety information (e.g., Table 3, quote ii). This primarily involved communication between the sectors, such as sharing information and developing materials; however, cooperation sometimes occurred on frontlines. For example, in British Columbia, a KI discussed how OH was involved in spreading information and ensuring compliance with safety plans for COVID-19 (Table 3, quote i.). British Columbia, Ontario and Quebec KIs spoke about how collaboration allowed for improved COVID-19 guidelines at the intervention development stage, as it fostered collaboration and communication between parties that typically did not interact (including different departments within an organization). In Quebec, a KI mentioned two groups that managed recommendations for the public and workplaces together developed consistent guidelines for masks. Similarly, a British Columbia KI said OH grew closer to PH during the pandemic while they worked together on safety plans (Table 3, quote i.). In Ontario, KIs spoke about how PH considered work environment factors in COVID-19 outbreak inspections. Also in Ontario, and while not mentioned directly by our KIs, it is worth mentioning that OH collaborated indirectly with PH by reimbursing employers for three paid COVID-related sick days; however, this reached only a portion of the working population because of the many (e.g., gig) workers classified as self-employed and presenteeism among low-wage workers due to fear of job loss. In Table 3, we highlight strengths in collaborations and intersections reported by KIs.

There were no Alberta examples of collaboration or cooperation between PH and OH from our data. This may be because we were unable to recruit PH participants from Alberta, who might have contributed examples. It may also reflect what our other Alberta participants described; that a preventive, protective PH/OH approach in Alberta had regressed, with low support for unions, and degraded protections through Occupational Health and Safety legislation change.

### Weaknesses

In this section we present KIs identification of organizational issues that created weaknesses in protecting workers and the public. These fell into two areas: communication, and the structure of separate prevention and workers' compensation policy. We draw on KI insights to illustrate how compensation is administered separately from worker protection in two provinces and we suggest that this may indicate prioritizing business at the expense of PH/OH and public/worker health and safety.

#### Government messages

An initial challenge to communication was that PH messages to the public could independently come from different levels of government. Sub-provincial (regional or local) PH units allowed for population-targeted program delivery and interventions. In PH, regional authorities could issue varied edicts. For instance, one PH expert KI described how a specific sub-provincial PH unit might act differently than the province (Table 4, quote i.).

**Table 4:** Weaknesses: Key points identified from quotes about PH-OH pandemic response and KI province.

**Quote no**.	**Prov**	**Quote**	**Weaknesses and related challenge**
i	Ontario	“So although there is a provincial guidance document, there's also at each health department level …. you know, each area will have their medical officer of health kind of dictate what needs to be done for each area …. (P3, PH expert, Ontario).	Targeted local response to COVID-19 were important for PH measures; yet, this created differences that could be confusing (e.g., live and work in two different areas)
ii	Ontario	“I got a lot of questions from participants about refusing the work, and at the beginning it was –according to Occupational Health and Safety Act, we advised that … [for example, when] a worker refuses work in the workplace due to a fear of contracting COVID-19 because they are immunocompromised, and these circumstances should be considered when determining how to handle these events …[but] in general, just for a fear and not going to work—‘I refuse to go to work because I'm afraid somebody else have COVID' –…. it's not acceptable” (P2, OH expert, Ontario).	Workers were uncertain about COVID-19 policy and the right to refuse unsafe work Fear alone was not an adequate reason for work refusal
iii	Ontario	“…so if there were an issue that is happening between an employer and employee that is outside of the PH scope, they would have to call the Ministry of Labor. But for us [in PH,] if it's COVID related or … related to anything under the public health legislation (and both are the regulations under the Health Protection and Promotion Act), then it's under our purview. In essence, we would share [physical] spaces. So, [for] a long-term care facility under COVID[-19 outbreak]: we go in to complete inspection, prevention, and control audit if that facility happens to be in an outbreak. If that facility is having issues with their employees' labor-wise, then of course the Ministry of Labor would be on site to inspect and/or any other ministry inspectors.” (P4, PH expert, Ontario)	PH and OH might technically share space but their activities within that space differ according to their purview
iv	Alberta	“… and the assumption from most people that if it's an employment related issue it's going to be covered under this umbrella employment problem. And the reality is, clients have to call us or have to call five different governments [across federal and provincial] and wait on hold to get that information” (P18, Employment Standards expert, Alberta)	Difficult for workers to obtain help/guidance
v	Alberta	“… in Alberta, our WCB and our occupational health and safety legislation are separate …. different provinces will have them together–and that allows for initiatives looking at the WCB, which was the compensation side—it's the post-injury or incident– working hand in hand with the prevention side …. we need to look at prevention initiatives, right, and … my sense is that that doesn't happen in the same way in Alberta because these two pieces are separated.… my sense is that employers didn't want their books opened up, right; … they don't want to be held liable for the stats that they release showing, essentially, that they have an unsafe workplace. (P17, OH expert, Alberta)	OH prevention and compensation are not within same organization Lack of data on workplace health incidents blocks timely identification of OH issues
vi	Alberta	“One of the main [issues] is none of the legislative bodies talk to each other. So, Occupational Health and Safety doesn't talk to Employment Standards, doesn't talk to EI [Employment Insurance], doesn't really talk to Human Rights…. So there's really a lack of clarity in terms of the synchronization of supports and I would say that's just a general gap across the board is the legislative requirements” (P18, Employment Standards expert, Alberta)	OH and PH legislation lack common legal frameworks
vii	Ontario	“…the challenge that we have in health and safety, it's the legislation … you could see it with Amazon workplaces…. The Ministry [of Labor] visited so many times, but nothing happened because, from the Ministry [of Labor] level—inspectors can only inspect from occupational health and safety [legislation]” (P2, OH expert, Ontario).	Ontario OH inspectors are limited to OH law, which limits their response to COVID-19 issues
viii	Alberta	“…new legislation here is supporting—basically responsibilizing folks or gig workers for their own rights, so, ‘health and safety is up to you'…and so you see this downloading of responsibility onto folks.…from a health and safety standpoint we don't have something that is in Ontario legislation, which is the Office of the Worker Advisor … We don't have that. So, unless you have a union … you've got no one. …That's the other thing, right, is that, over the years, you've seen what used to be government departments or what was seen as priorities for government, you know, services being cut and then downloaded onto the not profit sector. … ” (P17, OH expert, Alberta)	Limited public protection for workers

This table provides KI quotes and describes key weaknesses identified from quotes about PH-OH pandemic response and KI province.

During the pandemic, the geographic boundaries of Ontario sub-provincial PH units defined where some measures were implemented. For instance, contact tracing operations were established at the local PH unit level. While local approaches are always needed for targeted PH interventions, in the context of COVID-19 they created neighboring areas with different regulations in place. Consistent messaging was important in this context: for example, an OH expert KI in Ontario said the Ministry of Labor received many questions from workers and always instructed people to refer to their local public health unit to see what was currently applicable in their area (Table 3, quote ii). They also noted it could be challenging for workers to obtain help or guidance from federal or provincial offices on related matters—for instance, regarding lost wages due to quarantine, or concerns about COVID as a workplace hazard.

#### Workers' compensation

From an OH perspective, one KI suggested that, because Alberta Workers' Compensation and OH prevention programs are run as separate organizational units, and because of employers' under-reporting of work accidents (to avoid workers' compensation claim costs), there was a disconnect between prevention in the workplace and illness management (Table 4, quote v.). This administrative division of the prevention and compensation OH components might have contributed to what some OH experts considered a relative lack of collaboration between OH and PH in Alberta, and to some extent in Ontario. For example, an Alberta KI described siloed legislation and workers not knowing where to obtain help. In British Columbia and Quebec, however, where KIs described OH and PH as more collaborative, both the prevention and workers' compensation arms of OH are operated under the same organizational umbrella.

In Ontario, KIs described that OH and PH shared physical space when PH entered workplaces; however, legislation and thus responsibility for health issues remain siloed between the OH and PH domains (Table 4, quote vii). The importance of clearly demarked responsibilities was realized during the pandemic. For example, one piece of guidance from the Ontario Ministry of Health clarified roles for: local PH units, the Ministry of Health, Ontario Health, Public Health Ontario, the Ministry of Labor, Training and Skills Development, and roles of employers (15). However, this document was created only in October 2021, almost 2 years after the declaration of the pandemic.

A potential gap for protecting workers' OH, as indicated by an OH expert in Ontario was that, while legislation included a broad description of employers having to take reasonable precautions to protect workers from COVID-19 exposure, the Ministry of Labor inspectors were limited to conducting inspections that focused on OH legislation and policy, which did not always overlap with PH considerations (Table 4, quote vii).

### Opportunities

Sectoral division of responsibility became problematic when COVID-19 crossed typical boundaries. Circumstances when PH authorities enter a workplace were raised by KIs. Some described tension around when COVID-19 is considered an occupational illness vs. worker exposure through or to the public. KIs also spoke about workers being uncertain of who was responsible for paying wages for time lost to quarantine or illness and employers being unsure of which authority to report illnesses to. Opportunities to bridge traditional sectoral boundaries, including opportunities for PH/OH collaboration and the management of infectious diseases were further identified by the KIs.

#### OH and PH boundaries: Who does what, when?

Several KIs suggested there was opportunity to consider health protection more holistically. In part, this was because many workers do not have traditional workplaces that could be inspected (e.g., self-employed workers, including gig workers). Some KIs also saw an opportunity to more widely consider health protections as inclusive of population health and social determinants of health, rather the current segregated PH and OH domains. For instance, an OH expert in British Columbia spoke about the importance of protection regardless of who was doing what (Table 5, quote i).

**Table 5:** Opportunities: Key points identified from quotes about PH-OH pandemic response and KI province.

**Quote no**.	**Province**	**Quote**	**Key points and opportunities**
i	British Columbia	“We [in OH] had much more [resources than PH] in terms of boots on the ground. So, we could go inspect places as a workplace. … We could note, ‘That …looks to be well over occupancy', and then …. share information with PH. And they could have their own inspectors go in, so there was that linkage, and again, so it ends up protecting PH even though that's not directly our mandate…. There's not much difference in anybody's mind…. So, it just has this kind of effect that … you call it workplace health and safety, but it ends up protecting others.” (P15, OH Expert, British Columbia).	OH resourced to inspect workplaces OH inspectors shared information with PH OH activities supported PH protection Opportunity for holistic view of protection
ii	Ontario	“…our environmental health team, the PH inspectors … can go out to … workplaces and go over a checklist and do an assessment and then identify the areas that they could tweak and make things a little bit safer. …they'll have a huge checklist of things that they will go through and then they'll see, ‘Okay, we had our break together for 30 min and our chairs are two feet apart.' So then they'll go through and make suggestions, ‘Okay, this is how you're going to decrease the risk of transmission amongst your staff while you're here. Make sure that the staff is wearing mask when they're having the patrons come and pick up their takeout.' The source of transmission is … identified during the case investigation and then the PH inspector will actually go to that facility or that restaurant and go through their checklist and see: Where are the areas of risk? and, what can be updated or corrected to decrease risk in the future?” (P3, PH expert, Ontario).	PH inspectors investigated the organization of work and workplace practices PH considered workplace areas of risk and provided strategies to mitigate risk Opportunity for involvement of OH inspectors in PH domains (e.g., during shortages due to PH emergencies)
iii	Ontario	“During the pandemic, when places need to be shut down, usually that's as a result of an outbreak of cases … and that would come from the [PH] Medical Officer of Health in that area because that's in response to an immense spread of that disease of public health significance being COVID. … So … they have two separate- not to say that they might not both be involved at the same time—but, for any workplaces that I've been aware of, Ministry of Labor has not been a key player in it. It's more the facility and PH … because it's all kind of around the disease” (P3, PH expert, Ontario)	PH came to workplaces in instances of diseases of PH concern Opportunity for involvement of OH inspectors in PH domains (e.g., during shortages due to PH emergencies)
iv	Ontario	“Just, all in all, we just need to expand those protections in terms of the sick time so people can be off when they're sick and just even wage coverage as well too. Is there enough [wage] coverage? Are there enough options? Because I mean with the work that I've done with COVID … you hear about workers coming into work and they're sick, but they should be home resting and recovering.” (P4, Ontario, Public Health Expert).	More social protections needed (e.g., paid sick days) to ensure sick workers can stay home when ill or quarantining Opportunity to consider social determinants of health and health in OH and PH policies
v	British Columbia	“So, we've been disappointed here in British Columbia in the PH authorities not demanding Employment Standards paid sick leave for all workers. They've all said, ‘Don't go to work if you're sick.' Well, it doesn't address the issue of my ability, my income security, my low wage, and my job security. I'm going to go to work if I need the money, right? That's been the past practice. …We've had it spreading particularly in the food processing industry where workers are working close together …. And all of these workers are low paid, racialized migrant workers, immigrant workers who desperately need jobs and income and are just not able to stay away from work. And again, it puts the onus for the protection of PH on these workers, right? The onus should not be on them [workers] to protect against the spread of disease” (P11, British Columbia, Employment Standards KI).	Unfair to require workers to stay home when ill without paid sick leave Final responsibility for PH protections fell onto workers Opportunity to consider social determinants of health and health in all policies
vi	Ontario	“… particularly in COVID, local PH has been working with the Ministry of Labor. For example, a workplace did have COVID cases. The workplace is required by law to inform WSIB and the Ministry of Labor and then COVID they are also required to inform their local PH unit. …. So, we've already been working together” (P4, Ontario, PH).	Informing multiple parties (Ministry of Labor, Workers' Compensation, and/or PH) presents opportunity for cross-government sector confirmation Opportunity for data sharing, verification of employer reporting by cross-referencing data
vii	Quebec	“As a PH authority, we have two laws. One law is the Occupational Health Act, which is the one that limits us in a certain way to go to all the workplaces at large. But we also have the PH law and that gives us a broader mandate. In COVID, we were not limited to these [priority workplace] sectors … [which] they don't even cover the majority of workers, so [protect] even less the more precarious sectors like services and maybe … retail. …So yes, we are limited to offer this [OH] service but we're not limited to act in an emergency like COVID, for example.” (P9, Quebec, Public Health KI).	COVID-19 led to OH addressing risk and prevention on a broader scale Policy allowed key actors to do what was required in the pandemic emergency
viii	Alberta	“The stats that have been talked about have been: ‘We're not … linking it to workplaces; this is community spread' … but …at the same time they're saying, ‘Our contact tracing is the shits'. So … the numbers that we are getting … supports [community spread]. You know: ‘Where'd you get it?', ‘I dunno', ‘Okay, must be community spread.' (P17, OH expert, Alberta).	Workplaces were defined as “community spread” Opportunity for advancing understanding of occupational determinants of health; not all workers had privilege of working from home Opportunity to consider variable risk settings (e.g., small business office vs. cashier in large grocery store)

This table provides KI quotes and describes key opportunities identified from quotes about PH-OH pandemic response and KI province.

We found that some boundaries created an artificial distinction between workplaces and PH, given that workplaces were a site of transmission for COVID-19, and that PH issues (e.g., community spread) affected front-line and essential workers. Workplaces with a public-worker interface were particularly illustrative of how workplace and PH issues intersected. For example, a PH KI in Ontario explained that the presence of COVID-19 in the workplace determined when there would be PH involvement at workplaces (Table 4, quote ii.). In Alberta, some KIs described a historically weak OH sector as having had adverse system-level consequences during the pandemic. For instance, one KI described how weaknesses in COVID-19 data collection led to failures in identifying workplace spread (Table 5, quote viii). OH expertise was also described as directly relevant to COVID challenges with broad prevention approaches including the hierarchy of controls (e.g., engineering controls; Table 6, quote ii), evaluating occupancy levels (Table 6, quote i), and specific exposure control measures, such as ventilation (Table 6, quote ii).

**Table 6:** Threats: Key points identified from quotes about PH-OH pandemic response and KI province.

**Quote no**.	**Province**	**Quote**	**Threats and failures**
i	Alberta	“There's been a big push in occupational health and safety circles and PH [regarding] … ventilation. This thing this … really downplayed, the airborne stuff, because no one wants to talk about it. … When you talk PH messaging, which is: wear a mask; remain far apart; don't do indoor gatherings. Well, no one's talking—why don't you do indoor gatherings? [laughs] …. It's because … the studies shows that your in-home ventilation systems are not capable of dealing with this thing if it's airborne. …. It also calls into account … all our infrastructure is aging, right. We haven't even dealt with… asbestos yet, and now you're asking us [laughs] to deal with [COVID-19]?.... The confusing stuff that's coming out… sometimes they'll be taken as gospel because it supports existing public policy or it's cheaper. But you won't extend the same amount [of support] to, like, there's emerging stuff here from multiple sources on the role of ventilation … or on the fact this is airborne.” (P17, OH expert, Alberta).	Failure of PH authorities to acknowledge transmission as airborne/keep up with science
ii	British Columbia	“So, you know, initially, when everybody was kinda freaking out about this stuff [laughs], the question … was …‘what kind of masks do we need?'…. And you …. look at hierarchy of controls …. Before you can start thinking about masks, you've got to … [make] sure people don't come to work if they're sick in the first place. You know, think about putting barriers in place as engineering controls; you've got to think about distancing as an administrative control. And *then* we'll talk about masks. And … PH was very supportive of that and thought that was aligned with their thinking on things … [but] they thought more about ‘layers of protection'. … Now we have something called ‘the hierarchy of controls' that has been out there in people's minds a little bit more than maybe it had been in the past.” (P15, OH Expert, British Columbia).	Failure to act on first line of defense which is eliminating risk of transmission (i.e., not coming to work sick)
iii	Ontario	“If you're a immunocompromised worker, you can make use of the human rights code; if you're dealing with occupational health and safety, you can make use of OSHA, you know; if you're unionized, which… is really not the case with gig workers, but you could use your collective agreement … so there's this kind of, like, unending amount of kind of legal tools that could be used. But … if the government's not willing to use them, it's kind of all for naught. And … it would have been perfectly feasible for the Ministry of Labor to do an occupational health and safety blitz, early on in the pandemic for essential workers (P5, Employment Standards expert, Ontario).”	Under-enforcement of OH laws
iv	Ontario	“…the system is, I would say … outdated for the current way employment is structured in Ontario. It still presumes essential factories and shopfloors where all the workers are [located] and you can inspect them at all times from 9 a.m. to 5 p.m. when it's not really the way these things work anymore (P6, OH expert, Ontario).	OH inspection approaches failed to keep pace with labor market realities
v	Ontario	“For example … a trucking company where the truck driver did a cross-border drive and at the border … they tested positive. And so, what are the next steps for the company and what are the next steps for the driver, right? So, in that case the cab was treated as a workplace” (P4, PH expert, Ontario)	PH faced difficulty addressing COVID-19 in various work types

This table provides KI quotes and describes key threats identified from quotes about PH- OH pandemic response and KI province.

When workplace outbreaks occurred, COVID-19 was generally considered by PH units as solely a PH issue. However, the situations reported by KIs indicate there may be an opportunity to bring OH authorities on board to assist with workplace issues. An additional reason for involving OH in workplace outbreaks during a pandemic is to alleviate the high demands on PH staff. Involving OH in some aspects of outbreaks in workplace investigations could draw on existing skills and free up PH to conduct other activities (e.g., vaccination clinics). Jointly addressing prevention and compensation presented a potential opportunity for cooperation as a workplace outbreak inspection could not only allow for preventive measures, but also provide opportunity for assessing the likelihood of the disease being considered an occupational-acquired disease. Extended cooperation presented further potential for support; for instance, by allowing OH to be involved in contact tracing for workers who test positive to COVID-19.

Cooperation among OH and PH occurred at a provincial level. Cooperation at a federal level was not apparent in our interviews. This may be because the federal government is largely seen as responsible for federal employees and federally regulated industries, while other industries fall under provincial OHS legislation. Another consideration is that most policy is at the provincial level, although we consider the federal Employment Insurance as an OHS protective policy.

##### Right to refuse unsafe work

In Alberta, new OH legislation took effect in 2021, which defined a legitimate “undue hazard” for workers who refuse to work due to safety concerns as something that poses a “serious and immediate health and safety threat,” such as sudden infrastructure collapse, broken equipment, or gas leaks (16). For respiratory viruses, if employers have taken “reasonable precautions,” such as barriers or space for physical distance, implemented sick leave policy, screening programs, or rules for wearing masks, the government explicitly stated, “If your employer has implemented reasonable controls to address the risk of respiratory viruses, it's unlikely that there will be an undue hazard under OH legislation” (16). In our view such situations may provide an opportunity for OH and PH to work together when a worker is concerned about the hazards of work and reasonable controls. As infectious disease experts, PH could be consulted by OH when there is doubt about the risk level, particularly as Alberta equated workplace transmission with community spread thereby dismissing elevated risk associated with workplace exposure. PH data (e.g., hazard ratios) in local areas could also be used as a baseline measure for undue hazard in the workplace. While this approach may present challenges in smaller and medium workplaces, drawing on information from large workplace may provide an opportunity to estimate workers' levels of risk, compared to community levels of risk.

In Ontario, differences occurred regarding what policy applied to a situation or what party was responsible. This especially created confusion in workplaces with a public interface, such as retail stores. For example, an Ontario OH KI (P2) noted that owner-operators who took deliveries or had customers pick up orders may not have developed workplace safety plans.

Workplace-based outbreaks sometimes drew in three authorities. For instance, in Ontario, the Ministry of Labor, workers' compensation, and local PH units each needed to be informed of disease outbreak situations. An opportunity in this case may be a shared-data system, which could serve as a cross-sectoral tool for OH and PH units as they could cross-reference data and follow up with employers who only reported to a single sector. A shared data system could also reduce the reporting burden on employers and support evaluation of OH and PH activities. Notably, as all occupational diseases are supposed to be reported to both the Ontario Ministry of Labor, Training and Skills Development and to workers' compensation, shared data systems might ensure that each organization received information reported to the other. Overall, a shared data system represents a major opportunity that would have benefits extending beyond COVID-19.

### Threats

#### Policy not effective

A few KIs spoke about the importance of ensuring sick workers were enabled to remain off work when unwell; however, some KIs noted that some worker populations could not afford to remain home and lose wages, and the employment and social security systems failed to fully compensate such workers for lost income. Ensuring workers were resourced (e.g., sick pay or lost wages benefits) to stay home when ill or isolating was a key strategy to slow the spread of COVID-19. Workers who continued to attend work while sick or in quarantine posed a threat to containing disease transmission. Paid days to quarantine during mandatory isolation were not always provided, especially for low-wage or other precarious workers (Table 5, quote v, Table 6, quote ii).

KIs noted various shortcomings in multisectoral collaboration sometimes resulted in less robust PH measures. Multiple KIs made reference to the idea that both OH and PH policies had not kept pace with the nature of work or science. For instance, one Alberta OH expert described how OH ventilation concerns did not receive the same sort of attention as other issues that fell within a more conventional PH evidence paradigm (e.g., Table 6).

Misalignment between OH and other authorities sometimes blunted the effect of OH mandates. For instance, when PH mandates required COVID-19-infected workers to stay home, employment standards legislation did not ensure that these workers could afford to take sick leave. Similarly, an employment standards KI described how OH legislation for workers' health protection existed but was not well enforced (Table 6, quote iii). Although the threats of a lack of enforcement were exposed by COVID-19, these concerns were well-noted prior to the pandemic. A 2019 report by the Auditor General of Ontario found the Ministry of Ontario, Labor and Skills Development found that 72% of businesses were uninspected. Of the 28% that were inspected, only one percent were proactive inspections (i.e. not in response to reported hazards) (17).

Even within government ministries, the application of policy to the COVID-19 situation created confusion and inconsistences, especially in relation to non-standard workplaces. For example, an Ontario OH expert described how the OH system missed large sections of the workforce that did not fit the setup of standard workplaces (Table 6, quote iv). Indeed, the lack of a physical workplace for some workers made it difficult for PH to disseminate prevention materials or for OH inspectors to conduct inspections. Non-static workplaces posed a further policy application challenge, as per the example a truck driver in Table 6, quote v).

Another interesting difference across provinces was how recognition of COVID-19 as an occupational illness differed. For instance, Alberta was the only province within our sample that did not consider COVID-19 as a presumed workplace-acquisition for some occupations, with the exception of healthcare workers. In Alberta, the workers' compensation board policy updated March 24, 2022 (18) was that every claim must be individually adjudicated whereas, in other provinces, blanket presumptions existed about workplace acquisition of COVID-19 for certain sectors, such as grocery or retail. Although not presumptive of workplace acquisition, Alberta's policy allowed case managers to “take into consideration” workplace acquisition on a case-by-case basis. Still, all jobs with greater risk were not taken into consideration, as the policy excluded, for example, grocery store clerks. For these workers, Alberta WCB stated that COVID-19 is not considered workplace acquired because: “You are in contact with many people but not specifically with infected shoppers. This is considered casual contact” (18). As one KI pointed out (Table 6, quote i) this position failed to acknowledge that workers who are in public-facing positions logically have greater exposure to the virus than those who do not interact with the public.

Taken together with other examples (e.g., Alberta's definitions of undue hazard under OH legislation and denial of work refusals), the Alberta compensation board's normalization of workers' having greater exposure risk *via* elevated contact with the public points to a larger issue. As some KIs indicated, there appeared to be a tendency in some jurisdictions to minimize state responsibility to the detriment of PH and OH protection for workers.

## Discussion

This aim of this paper was to examine how OH and PH coordinated with each other during the pandemic. We found that, while cooperation occurred between PH and OH to varying degrees, organizational factors such as sectoral policy and practice boundaries appeared to lead to siloed approaches and constituted a barrier to optimal communication and fulsome collaboration. Using SWOT analysis highlighted that the division between PH and OH did not allow for consideration of COVID-19 risks in all workplace situations. Such organizational and policy appeared to leave blind spots in worker protection. In this discussion, we consider how protection and gaps in the wake of government levels and sectoral divides might be addressed. We also examine opportunities to facilitate workers' health and safety from a broader, multisectoral lens.

### Protection and gaps

The separate OH and PH architecture in Canada contributed to some weaknesses in the COVID-19 response. Our data suggested that there are several opportunities for mutually supportive interaction and collaboration among the two authorities. As far back as 2004, multisectoral communication was noted as important in Canada when health issues cross geographic boundaries:

“The issue of externalities and spillovers is closely linked to the primary reason why governments need to interact in public health. Threats to health produced in one region have the potential to spread and cause harm to individuals who live in other regions. For example, if one province chooses not to immunize its children against a certain condition, then the effectiveness of the immunization programs in other parts of Canada can be undermined” (p. 410) (19).

Our data support other findings that, despite OH legislation intended to protect Canadian workers, including *via* the right to refuse unsafe work and a right to information, there have been numerous failures in practice with respect to COVID-19 (20). Lippel (20) noted that, in Ontario, workers were required to work even when personal protective equipment was unavailable and health information was not forthcoming, and the Ontario Ministry of Labor appeared to systematically deny work refusals and that this “underreporting” of workers' compensation claims appeared to have been occurring for COVID-19. Our analysis adds that the division of PH and OH responsibilities did not fully consider the situated reality of workers in the context of infectious disease, such as COVID-19. Our findings suggest PH/OH segregation may have overlooked some workplace OH concerns as health issues for PH consideration. For example, recent investigative journalism found that Ontario's ‘essential' workers in manufacturing accounted for more workplace COVID deaths than any other sector, including health care (21).

An ethical issue emerges in consideration of public health orders requiring sick workers to stay home but not requiring income support compensation for those workers. This violates the principle of reciprocity, which calls for society to provide options to support individuals to “discharge their duty” when a public health measure burdens them (22). In the case of sick workers, wherein it is their public health duty to remain home when sick, the principle of reciprocity indicates that they should be financially compensated so they are able to afford to stay home and are not forced to go to work and expose others as a result of their own financial need.

The pandemic illustrated that PH and OH have complementary purposes that can be beneficial for protecting the health of populations during a pandemic. KIs described some instances where PH experts consulted with OH to devise recommendations for different workplace sectors; however, there is also room for this knowledge exchange to flow in the other direction: from OH to PH. OH expertise in airborne exposures is rich in tools and practices for measuring various substances and the appropriate protective measures (23, 24). For instance, OH experts did not support the notion of a strictly droplet spread of COVID-19, but rather, that particulate occurred in various sizes including airborne particles (25). Many OH experts recommended the use of fitted N95 masks as useful control measures early in COVID-19—masks that PH still did not recommended for most settings even two years into the pandemic (25).

Collaboration becomes especially important when staff surge capacity is required (26). PH-OH collaboration could also be a viable option. For instance, our data showed that the British Columbia labor ministry had inspectors already visiting workplaces (Table 3, quote i). Providing OH inspectors with training and protocols for managing implementation of safety plans and some limited and appropriate workplace-inspection components of PH investigations was a missed opportunity that might have freed up more PH workers. While some support for PH could be facilitated in this manner, identified PH concerns (e.g., outbreak scenarios) would still require PH investigation.

### Summary of results and actionable policy items

The separation of PH and OH organizationally and legislatively meant lost opportunities for responding to COVID (e.g., failure to draw on OH expertise around airborne controls/policies). Based on KI information both OH and PH policies and organization were not sufficient to protect non-standard workplaces and workers (e.g., mobile workers). While OH inspectors were physically present in workplaces, they could not help prevent disease transmission due to limits in legislative scope, and there was no evidence of communication to engage them in that role by PH, except in British Columbia. Despite barriers, PH and OH collaborations that did occur generated beneficial policies and actions (e.g., unified mask requirements for community and workplace settings in Quebec).

The physical presence of OH in workplaces offers PH an opportunity for enhanced preventive actions, particularly during emergency circumstances where OH may be able to provide PH surge capacity. Collaboration between OH and PH practitioners leads to beneficial policies and should be supported (e.g., *via* interpersonal networking, or formalizing multisectoral activities).

### Study strengths and limitations

A strength of this study was the comparative examples from four Canadian provinces. This allowed for observation of a range of OH and PH activities during COVID-19 and triangulation of themes. A further strength of this study was the multi-sectoral knowledge of our KIs that allowed us to probe various aspects of PH/OH regarding the protection of workers. A limitation of this study was that our sample in each area of expertise for each province was small and we were unable to obtain PH participants from Alberta. Therefore, our findings should not be considered representative of every jurisdiction or generalizable. Nevertheless, our sampling strategy allowed for data generation that raises useful propositions. Further, as a qualitative study our study was able to access contextualized accounts of policy situations.

## Conclusion

The COVID-19 pandemic response saw multisectoral OH-PH communication and collaboration become important for improved worker and PH measures and operational function. As OH and PH policies are updated across Canada, more cooperation among these authorities may overcome communication weaknesses. Our study suggests that there is opportunity in cooperation: OH and PH stakeholders found value in, and may support the continuation of, new productive relationships. Overall, there is also opportunity for more coordination of PH and OH measures; for instance, the precautionary principles from OH and the upstream preventive measures of PH each offer upstream opportunity to evaluate risk. We conclude that a collaborative multisectoral approach with mutual PH and OH supports could strengthen Canada's response to the risk of COVID-19 and potentially other health risks.

## Data availability statement

The original contributions presented in the study are included in the article/supplementary material, further inquiries can be directed to the corresponding author.

## Ethics statement

The studies involving human participants were reviewed and approved by the University of Waterloo Office of Research. Written informed consent for participation was not required for this study in accordance with the national legislation and the institutional requirements.

## Author contributions

EM, SEM, and SBM conceived of and designed the study. Interviews were conducted by PH and all authors contributed to data analysis. The first draft of the manuscript was written by PH and all authors commented on previous versions of the manuscript. All authors read and approved the final manuscript.

## Funding

This study was funded by a Canadian Institute for Health Research Operating Grant: SARS-CoV-2 variants supplement stream grant (Grant # VS1-175546).

## Conflict of interest

The authors declare that the research was conducted in the absence of any commercial or financial relationships that could be construed as a potential conflict of interest.

## Publisher's note

All claims expressed in this article are solely those of the authors and do not necessarily represent those of their affiliated organizations, or those of the publisher, the editors and the reviewers. Any product that may be evaluated in this article, or claim that may be made by its manufacturer, is not guaranteed or endorsed by the publisher.
